# Supplementary material for: Are Experts Well-Calibrated? An Equivalence-Based Hypothesis Test
Source: Entropy (Basel). 2022 May 27;24(6):757. doi: 10.3390/e24060757 (PMC9222732; doi:10.3390/e24060757)
Supplement: Supplementary file 1 [file entropy-24-00757-s001.zip › File S1. R program for simulating and analyzing data.pdf]

R program for simulating and analyzing data

```
# Number of repeats of simulation at each combination of true level of
# coverage of intervals and the number of elicited intervals.

nrep <- 25

# Assigned true levels of coverage of elicited intervals to be tested
# for experts' calibration at considered levels of intended coverage
# probabilities.

true_cov_levels <- c(cov1, cov2, cov3, .., )
n_coverage <- length(true_cov_levels)

# Average proportions of experts who are identified as well-calibrated
# from the equivalence test

avg_prop_equivalence <- vector(length = n_coverage)

# Average proportions of experts who are identified as well-calibrated
# from the direct test

avg_prop_equal_hit <- vector(length = n_coverage)

# Setting seed values

set.seed(123)

for(i in 1 : n_coverage)
{
avg_prop_equivalence[i] <- 0
avg_prop_equal_hit[i] <- 0

# Number of experts who are identified as well-calibrated from the
# equivalence test

num_equivalence <- vector(length = 100000)
```

```
# Number of experts who are identified as well-calibrated from the
# direct test

num_equal_hit <- vector(length = 100000)

# Proportions of experts who are identified as well-calibrated from
# the equivalence test

prop_equivalence <- vector(length = 100000)

# Proportions of experts who are identified as well-calibrated from
# the direct test

prop_equal_hit <- vector(length = 100000)

for(m in 1 : 100000)
{
num_equivalence[m] <- 0
num_equal_hit[m] <- 0
prop_equivalence[m] <- 0
prop_equal_hit[m] <- 0

# Number of intervals contain true values

x <- vector(length = nrep)

# Computed hit rates

sample_prop <- vector(length = nrep)

for(j in 1:nrep)
{
sample_prop[j] <- 0
x[j] <- 0
x[j] <- rbinom(1, n, true_cov_levels[i])
sample_prop[j] <- x[j]/n

if(sample_prop[j] == conf_level)
{
num_equal_hit[m] <- num_equal_hit[m] + 1
}

if ((C1 < x[j]) & (x[j] < C2))
{
num_equivalence[m] <- num_equivalence[m] +1
}
```

```
else if(C1 == x[j])
{
u1 <- runif(1, min = 0, max = 1)
if (u1 < gam1)
{
num_equivalence[m] <- num_equivalence[m] +1
}
}

else if( C2 == x[j])
{
u2 <- runif(1, min = 0, max = 1)
if (u2 < gam2)
{
num_equivalence[m] <- num_equivalence[m] +1
}
}

else
{
num_equivalence[m] <- num_equivalence[m]
}

}
 prop_equivalence[m] <- num_equivalence[m]/nrep
 prop_equal_hit[m] <- num_equal_hit[m]/nrep
}

avg_prop_equivalence[i] <- mean (prop_equivalence)
avg_prop_equal_hit[i] <- mean (prop_equal_hit)
}
result <- data.frame(true_cov_levels,
        avg_prop_equivalence, avg_prop_equal_hit)

result
```

Note: The above program can be adjusted to compute the respective values of the average proportions of experts who are correctly identified as well-calibrated from the non-randomized equivalence test by excluding the last two steps (7 and 8) of the Wellek's equivalence test described in a supplementary material.
