# Supplementary material for: Are Experts Well-Calibrated? An Equivalence-Based Hypothesis Test
Source: Entropy (Basel). 2022 May 27;24(6):757. doi: 10.3390/e24060757 (PMC9222732; doi:10.3390/e24060757)
Supplement: Supplementary file 1 [file entropy-24-00757-s001.zip › File S2. Wellekí»s equivalence test.pdf]

Wellek's equivalence test

We use the notation, formulations, and the details about the exact version of the equivalence test of a single binomial proportion presented in Wellek (chapter 4 in [13]). Some of the material will simply be repeated (rather than just referred to) for the sake of completeness.

The binomial distribution can be considered as a sum $T = \sum_{i=1}^{n} X_i$ of n mutually independent Bernoulli random variables; $X_1, X_2, ..., X_n$, with two outcomes, say *success* and *failure* with probability p and $1-p$, respectively. The probability mass function of the distribution of T is:

$$f(T = t; n, p) = \binom{n}{t} p^t (1-p)^{n-t}, t \in \{0, 1, 2, ..., n\}. \quad (1)$$

Suppose that an unknown population proportion of success P is required to be statistically tested for equivalence with a reference value P_r. The statistic of interest in defining the equivalence test of a single binomial proportion is the number of successes T out of a certain n number of Bernoulli trials. Let us assume that equivalence can be claimed if P remains between $P_1 = P_r - \epsilon_1$ and $P_2 = P_r + \epsilon_2$, where an acceptable margin of deviation around the reference value is allowed in the test. P_1 and P_2 can be made symmetric around P_r by taking $\epsilon_1 = \epsilon_2 = \epsilon$, unless specific, different values are preferred for ϵ_1 and ϵ_2, which will be context dependent.

An equivalence test includes two null hypotheses and a single alternative hypothesis, from which equivalence can only be claimed by rejecting both null hypotheses. Furthermore, allowing an acceptable margin of deviation ϵ around the reference value P_r leads to a claim of equivalence if P remains between $P_1 = P_r - \epsilon$ and $P_2 = P_r + \epsilon$. Therefore, the alternative hypothesis is defined as $P_1 < P < P_2$ and the two null hypotheses should be defined as $0 < P \leq P_1$ and $P_2 \leq P < 1$. Formally:

$$\begin{aligned} H_0 :& 0 < P \leq P_1 \text{ or } P_2 \leq P < 1 \\ H_1 :& P_1 < P < P_2, \text{ where } (0 < P_1 < P_2 < 1). \end{aligned}$$

The following set of rules define a uniformly most powerful level α test that can be defined by the following set of rules:

1. Rejection of H_0 for $C_\alpha^1 < T < C_\alpha^2$
2. Rejection with probability γ_α^1 for $T = C_\alpha^1$

3. Rejection with probability γ_α^2 for $T = C_\alpha^2$

4. Acceptance for $T < C_\alpha^1$ (or $T > C_\alpha^2$)

It is worth mentioning that all constants C_1, C_2, γ_1, and γ_2 depend on the values of n, P_1, P_2 The following equations (eq. 2), together with the probability mass function $f(T = t; n, P)$ of the binomial random variable T (eq. 1), and an iterative algorithm can be used to derive these constants.

$$\sum_{t=C_1+1}^{C_2-1} f(T=t;n,P_1) + \sum_{\nu=1}^{2} \gamma_\nu \, f(T=C_\nu;n,P_1) = \alpha \quad (2)$$
$$\sum_{t=C_1+1}^{C_2-1} f(T=t;n,P_2) + \sum_{\nu=1}^{2} \gamma_\nu \, f(T=C_\nu;n,P_2) = \alpha;$$
$$0 \leq C_1 \leq C_2 \leq n, \quad 0 \leq \gamma_1, \gamma_2 < 1.$$

The iterative algorithm necessary to derive a solution for C_1, C_2, γ_1, and γ_2 is as follows chapter 4 in [13].

1. Select an initial value C_1^0 for the lower bound of the rejection region knowing that it is greater or equal to the correct value C_1.

2. Keeping C_1^0 fixed, find the largest integer $C_2^0 > C_1^0$ such that the probability of observing T to take on its value in the closed interval $[C_1^0+1, C_2^0-1]$ does not exceed α, neither for $P = P_1$ nor for $P = P_2$.

3. Consider equation 2 as a system of linear equations in the two unknowns γ_1 and γ_2 and compute its solution γ_1^0, γ_2^0.

4. Test whether the condition $0 \leq \gamma_1^0, \gamma_2^0 < 1$ is satisfied. If so, a solution of the full system in equation 2 is found and can be given as $(C_1, C_2, \gamma_1, \gamma_2) = (C_1^0, C_2^0, \gamma_1^0, \gamma_2^0)$. If not, reduce C_1^0 by 1 and repeat steps (2) and (3).

The inclusion of the γ_1 and γ_2 rejection probabilities at the border values $T = C_1$ and $T = C_2$ in the equation 2 implies that the above discussed level α test of the equivalence test is defined as a randomized test. The equivalence-based hypothesis testing procedure can be implemented to test the calibration of a given expert at a certain intended level of coverage probability as follows:

1. Consider the intended level of coverage probability as the reference value P_r.

2. Define $P_1 = P_r - \epsilon$ and $P_2 = P_r + \epsilon$ based on a fixed, chosen value of ϵ.

3. Compute the limits of the rejection region C_1 and C_2 at a given number of elicited intervals (n).

4. Observe the number of intervals containing true values (x) as a random variable for the expert.

5. If $x < C_1$ OR $x > C_2$, then do not reject the null hypotheses.

6. If $x > C_1$ AND $x < C_2$, then reject the null hypotheses and conclude the equivalence indicting that the expert is well-calibrated at the intended coverage probability P_r.

7. If $x = C_1$, then generate a uniform random number u. If $u < \gamma_1$, then reject the null hypotheses and conclude the equivalence. Otherwise do not reject the null hypotheses.

8. If $x = C_2$, then generate a uniform random number u. If $u < \gamma_2$, then reject the null hypotheses and conclude the equivalence. Otherwise do not reject the null hypotheses.

Note: When $x = C_1$ or $x = C_2$ the test is inconclusive. In such a situation, a uniform distributed random number u should be generated and compared with γ_1 or γ_2 appropriately to make a conclusion.

References

[13] Stefan Wellek. *Testing statistical hypotheses of equivalence and noninferiority.* Chapman and Hall/CRC, 2010.
