# Supplementary material for: Are Experts Well-Calibrated? An Equivalence-Based Hypothesis Test
Source: Entropy (Basel). 2022 May 27;24(6):757. doi: 10.3390/e24060757 (PMC9222732; doi:10.3390/e24060757)
Supplement: Supplementary file 1 [file entropy-24-00757-s001.zip › File S3. Wellek's R program.pdf]

Wellek's R program

```
bi1st <- function(alpha,n,P1,P2)        {

K <- trunc(n/2)
indiP1K <- 0
indiS6 <- 0


if (2*K >= n && P2 == (1-P1))
 { P1K <- pbinom(K,n,P1) - pbinom(K-1,n,P1)
   if (P1K >= alpha)
    { C1 <- K
      C2 <- K
      gam1 <- alpha/P1K
      gam2 <- gam1
      POWNONRD <- 0
      POK <- pbinom(K,n,.5) - pbinom(K-1,n,.5)
      POW <- gam1 * POK
      indiP1K <- 1  } }

if (indiP1K != 1)
{
P0 <- (P1+P2) / 2
K1 <- max(trunc(n*P1),1)
K2 <- max(trunc(n*P0)-2,K1-1)

repeat
{
FBINP1C1 <- pbinom(K1-1,n,P1)
alpha1 <- 0
FBINP2C1 <- pbinom(K1-1,n,P2)
alpha2 <- 0

while(max(alpha1,alpha2) <= alpha)
  {  alpha1 <- pbinom(K2,n,P1) - FBINP1C1
     alpha2 <- pbinom(K2,n,P2) - FBINP2C1
     K2 <- K2 + 1  }
```

```
K2 <- K2 - 2
if(K2 < K1)
  { INCL <- 1
    INCR <- 1  } else

   { K1 <- K1 + 1
     INCL <- 0
     INCR <- 1 }

repeat
 {
alpha1 <- pbinom(K2,n,P1) - pbinom(K1-1,n,P1)
alpha2 <- pbinom(K2,n,P2) - pbinom(K1-1,n,P2)
delalph1 <- alpha - alpha1
delalph2 <- alpha - alpha2
b11 <- pbinom(K1-1,n,P1) - pbinom(max(K1-2,0),n,P1) * sign(1+sign(K1-2))
b12 <- pbinom(K2+1,n,P1) - pbinom(K2,n,P1)
b21 <- pbinom(K1-1,n,P2) - pbinom(max(K1-2,0),n,P2) * sign(1+sign(K1-2))
b22 <- pbinom(K2+1,n,P2) - pbinom(K2,n,P2)

gam1 <- (b22*delalph1 - b12*delalph2) / (b11*b22 - b12*b21)
gam2 <- (b11*delalph2 - b21*delalph1) / (b11*b22 - b12*b21)


if ((min(gam1,gam2)<0 || max(gam1,gam2) >= 1) && INCL == 0 && INCR == 1)
   { K1 <- K1 - 1
     K2 <- K2 - 1
     INCL <- 1
     INCR <- 0  } else

if ((min(gam1,gam2)<0 || max(gam1,gam2) >= 1) && INCL == 1 && INCR == 0)
   { K2 <- K2 +1
     INCL <- 1
     INCR <- 1  } else

if ((min(gam1,gam2)<0 || max(gam1,gam2) >= 1) && INCL == 1 && INCR == 1)
   { K1 <- K1 +1
     break    } else

   { indiS6 <- 1
     break    }   }

if (indiS6 == 1)
    break          }
```

```
C1 <- K1 - 1
C2 <- K2 + 1
fbinpoc1 <- pbinom(C1,n,P0)
BO1 <- fbinpoc1 - pbinom(max(C1-1,0),n,P0) * sign(1+sign(C1-1))
fbinpok2 <- pbinom(K2,n,P0)
BO2 <- pbinom(C2,n,P0) - fbinpok2
POWNONRD <- fbinpok2 - fbinpoc1
POW <- gam1*BO1 + POWNONRD + gam2*BO2
if (C1 == C2) POW <- POW/2                    }

cat(" alpha =",alpha,"   n =",n,"   P1 =",P1,"   P2 =",P2,"   C1 =",C1,
    "   C2 =",C2,"\n","gam1 =",gam1,"   gam2 =",gam2,"   POWNONRD =",POWNONRD,
    "   POW =",POW)            }
```

Try the EQUIVNONINF package in your browser
library(EQUIVNONINF)

help(bi1st)

Any scripts or data that you put into this service are public.

EQUIVNONINF documentation built on July 12, 2021, 5:08 p.m.
R Package Documentation
rdrr.io home
R language documentation
Run R code online
Browse R Packages
CRAN packages
Bioconductor packages
R-Forge packages
GitHub packages
We want your feedback!
Note that we can't provide technical support on individual packages.
You should contact the package authors for that.
Tweet to @rdrrHQ
GitHub issue tracker
ian@mutexlabs.com
Personal blog
